# Unlike Glycerophosphocholine or Choline Chloride, Dietary Phosphatidylcholine Does Not Increase Plasma Trimethylamine-*N*-Oxide Levels in Sprague-Dawley Rats

**DOI:** 10.3390/metabo12010064

**Published:** 2022-01-11

**Authors:** Bungo Shirouchi, Ayano Fukuda, Taiki Akasaka

**Affiliations:** 1 Department of Nutrition Science, Faculty of Nursing and Nutrition, University of Nagasaki, Siebold, 1-1-1 Manabino, Nagayo-cho, Nishi-Sonogi-gun, Nagasaki 851-2195, Japan; bn218030@sun.ac.jp; 2 Center for Advanced Instrumental and Educational Supports, Faculty of Agriculture, Kyushu University, 744 Motooka, Nishi-ku, Fukuoka 819-0395, Japan; akasaka@agr.kyushu-u.ac.jp

**Keywords:** phosphatidylcholine, glycerophosphocholine, choline chloride, TMAO, gut microbiota

## Abstract

Choline, betaine, and L-carnitine are transformed into trimethylamine (TMA) by gut microbiota, absorbed into the liver, and oxidized into trimethylamine-*N*-oxide (TMAO) by flavin-containing monooxygenases. Elevated TMAO levels may negatively affect human health. As phosphatidylcholine (PC) is the main source of dietary choline, its intake or PC-rich foods may be harmful to human health; however, quantitative comparative information among dietary choline compounds (PC, glycerophosphocholine [GPC], and choline chloride [CC]) regarding in vivo generation of TMAO is lacking. Here, we compared the effects of PC, GPC, and CC on plasma TMAO levels in rats. Furthermore, we investigated their effects on gut microbiota at the genus level. Dietary PC did not affect plasma TMAO levels, whereas dietary GPC and CC significantly increased them. At the genus level, plasma TMAO levels were significantly negatively correlated with relative abundances of *Anaerotruncus*, *Actinomyces*, *Enterococcus*, *Dialister*, *Clostridium* XIVa, and *Granulicatella*; they were significantly positively correlated with that of *Coprobacter*. Moreover, the relative abundances of *Anaerotruncus* and *Coprobacter* were found to predict plasma TMAO levels. Therefore, dietary PC, unlike GPC or CC, does not increase plasma TMAO levels in rats. Furthermore, several gut microbes are associated with changes in plasma TMAO levels in rats fed with choline compounds.

## 1. Introduction

**Figure 1:**
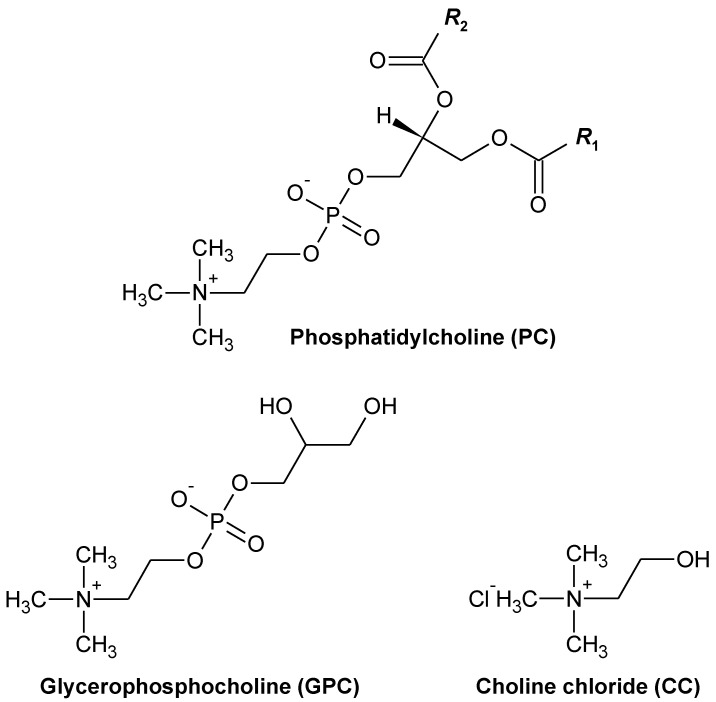
Chemical structures of phosphatidylcholine (PC), glycerophosphocholine (GPC), and choline chloride. “R” represents hydrocarbon groups.

Phospholipids (PLs) are constituents of cell membranes; they are present at various concentrations in lipid-containing foods. In humans, PL intake is estimated to be 3–6 g·d^−1^, which amounts to approximately 10% of the total dietary lipid intake [1]. Growing evidence indicates that dietary PLs have beneficial effects, including the prevention of dyslipidemia, fatty livers, and cardiovascular disease, compared with dietary triglycerides (TGs) [2,3,4]. Glycerophospholipids (GPLs), which are classified together with sphingophospholipids as PLs, are composed of hydrophobic (e.g., fatty acids) and hydrophilic (e.g., choline, ethanolamine, inositol, or serine) components; this composition may be responsible for their physiological function. Phosphatidylcholine (PC) constitutes the majority of dietary GPLs [1]; it is also an established source of choline, which is a water-soluble vitamin-like essential nutrient [5]. Numerous studies have suggested that dietary PC exerts a protective effect against cardiovascular disease (CVD) by improving the blood lipid profile [2,3,4]. In contrast with previous studies, independent of improving the blood lipid profile, a recent study has suggested that dietary PC may increase the risk of CVD through its metabolites, such as choline [6]. Dietary choline, betaine, and L-carnitine can all undergo catabolism by gut microbiota to form trimethylamine (TMA), which is absorbed and then rapidly oxidized into trimethylamine-*N*-oxide (TMAO) in the liver by flavin-containing monooxygenases (FMOs) [7,8,9]. Elevated circulating levels of TMAO are considered to have negative effects on human health; they are a risk factor for CVD, colorectal cancer, and chronic kidney disease [6,10,11,12]. Based on the above-mentioned studies into TMAO, the intake of PC or PC-rich foods has been suggested to be harmful to human health. On the other hand, choline in PC has been reported to be absorbed more efficiently than free choline [13,14], suggesting that choline in PC may be less susceptible to catabolism by gut microbiota than free choline. Taken together, the question as to whether dietary PC exerts a beneficial effect on CVD remains unclear. In addition, there is currently a lack of quantitative comparative information among dietary choline compounds (PC, glycerophosphocholine [GPC], and choline chloride [CC]) regarding the in vivo generation of TMAO (Figure 1).

The conversion of TMA by gut microbiota in the host is an important part of choline metabolism regarding in vivo TMAO generation. The key step in the microbial catabolism of choline is the cleavage of the C-N bond, which is catalyzed by the glycyl radical enzyme choline TMA-lyase (CutC) along with its activating enzyme (CutD), under an anaerobic environment. This results in the production of acetaldehyde and TMA [15,16]. The extent of the conversion of choline (and related substances) into TMA depends on the type of gut microbiota in the host. Therefore, although there is a growing understanding of microbial choline catabolism, it is difficult to obtain quantitative comparative information among the conversion of different dietary choline compounds into TMAO, via the oxidation of TMA, in human intervention studies.

Therefore, to explore the abilities of different types of choline compounds (PC, GPC, and CC) to be converted into TMAO, here we investigated whether plasma TMAO levels increased in rats that were fed these three types of choline compounds. In addition, we evaluated the effects of dietary intake on the gut microbiota of rats.

## 2. Results and Discussion

Here, to explore the abilities of different types of choline compounds (PC, GPC, and CC) to be converted into TMAO, we investigated whether plasma TMAO levels increased in rats that were fed three types of choline compounds. In addition, we investigated the effects of dietary intake on the gut microbiota of rats.

### 2.1. Effects of Dietary PC, GPC, and CC on the Growth and Metabolic Parameters in SD Rats

There was no difference in food intake, liver weight, weights of mesenteric and abdominal WATs, or soleus muscle weight (Table 1). The epididymal WAT weight in the CC group was significantly lower than that in the control group (Table 1), while the perirenal WAT weight in the GPC group was significantly lower than that in the control group (Table 1). In addition, the GPC and CC groups had significantly lower liver PL contents than the control group (Table 1). PL-derived choline is absorbed in the small intestine more efficiently than free forms of choline [13,14], resulting the former having a higher bioavailability. PC, which is synthesized from choline, is known to constitute the outer layer of the cell membrane lipid bilayer [5]. Therefore, changes in the morphometric and metabolic parameters of normal SD rats fed with GPC or CC may have contributed to the low bioavailability observed for choline derived from these sources.

### 2.2. Effects of Dietary PC, GPC, and CC on Plasma TMAO Levels and mRNA-rElated TMAO Generation in Livers of SD Rats

**Figure 2:**
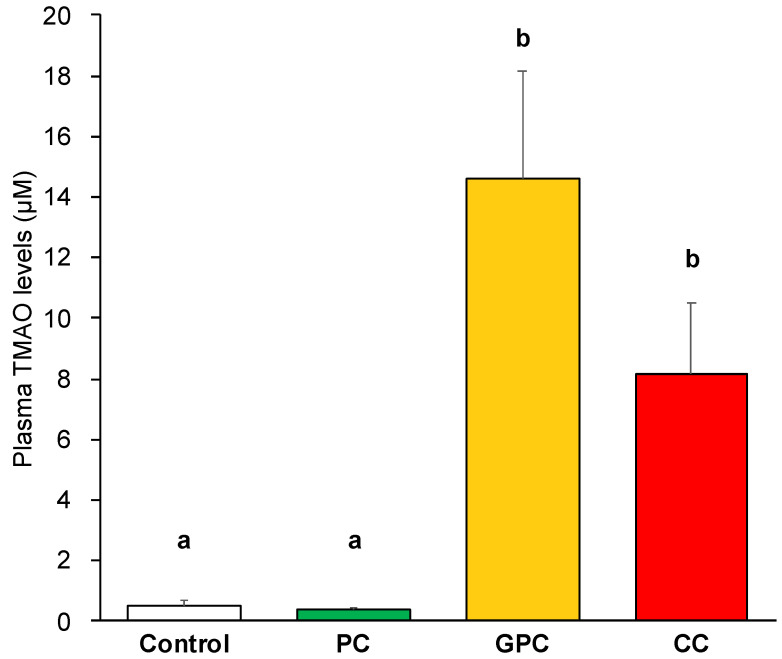
Effects of dietary choline compounds on plasma TMAO levels in rats. Values are expressed as means ± SEM (*n* = 5 per group). ^a,b^ Different letters show significant differences at *p* < 0.05 using one-way ANOVA followed by Tukey–Kramer multiple comparison post hoc tests.

Plasma TMAO levels were significantly higher in the GPC and CC groups than in the control and PC groups (Figure 2). As mentioned above, PL-derived choline is absorbed more efficiently in the small intestine than free forms of choline [13,14], suggesting that it is difficult to convert PC-derived choline into TMA in the large intestine, and that it does not increase plasma TMAO levels. Our results are supported by the findings of a previous study, which reported that choline bitartrate yielded a plasma TMAO incremental area under the curve (iAUC) that was three times greater than that obtained with no choline and PC in healthy men [17]. Our present study is the first to report that dietary GPC increases plasma TMAO levels, in addition to dietary CC. Using the online lipophilicity/aqueous solubility calculation software (ALOGPS 2.1) [18], here we compared the octanol-water partition coefficients (logP) of CC, GPC, and 1-parmitoyl-2-linoleoyl-*sn*-glycero-3-phosphocholine (which is the main molecular species of soybean PC), and their values were as follows: CC: −3.59, GPC: −2.56, and 1-parmitoyl-2-linoleoyl-*sn*-glycero-3-phosphocholine: 5.48. The large intestine has three primary functions: the absorption of water and electrolytes, the production and absorption of vitamins, and the formation and propulsion of feces toward the rectum for elimination [19]. Therefore, here we propose that dietary GPC and CC increase plasma TMAO levels by facilitating their transition to the large intestine, by means of their physicochemical properties (which are related to water behavior in the large intestine); they are then fermented by gut microbiota.

To further examine the effects of dietary choline compounds on plasma TMAO levels, we analyzed mRNAs related to TMAO generation in the liver. It has been reported that plasma TMAO levels shown a significant positive correlation (*R* = 0.80, *p* <0.001) with hepatic *Fmo3* mRNA levels in mice, and a trend toward positive association (*R* = 0.49, *p* = 0.07) with hepatic *FMO3* mRNA levels in humans [6]. Adult human liver mRNA exhibits high *FMO3* expression and extremely low *FMO1* expression [20]; in contrast, rat livers exhibit moderate FMO1 protein expression [20]. Based on this information, here we measured the mRNA levels of two *Fmo* isoforms (*Fmo1* and *Fmo3*) in the livers of SD rats. As shown in Table 1, dietary choline compounds did not affect hepatic *Fmo1* and *Fmo3* mRNA levels, suggesting that changes in plasma TMAO levels depend on the fecal amounts and plasma levels of TMA as a substrate; that is, they may vary due to the changes in the gut microbiota of rats.

### 2.3. Effects of Dietary PC, GPC, and CC on Gut Microbiota in SD Rats: Identification of Gut Microbes Associated with Changes in Plasma TMAO Levels

**Figure 3:**
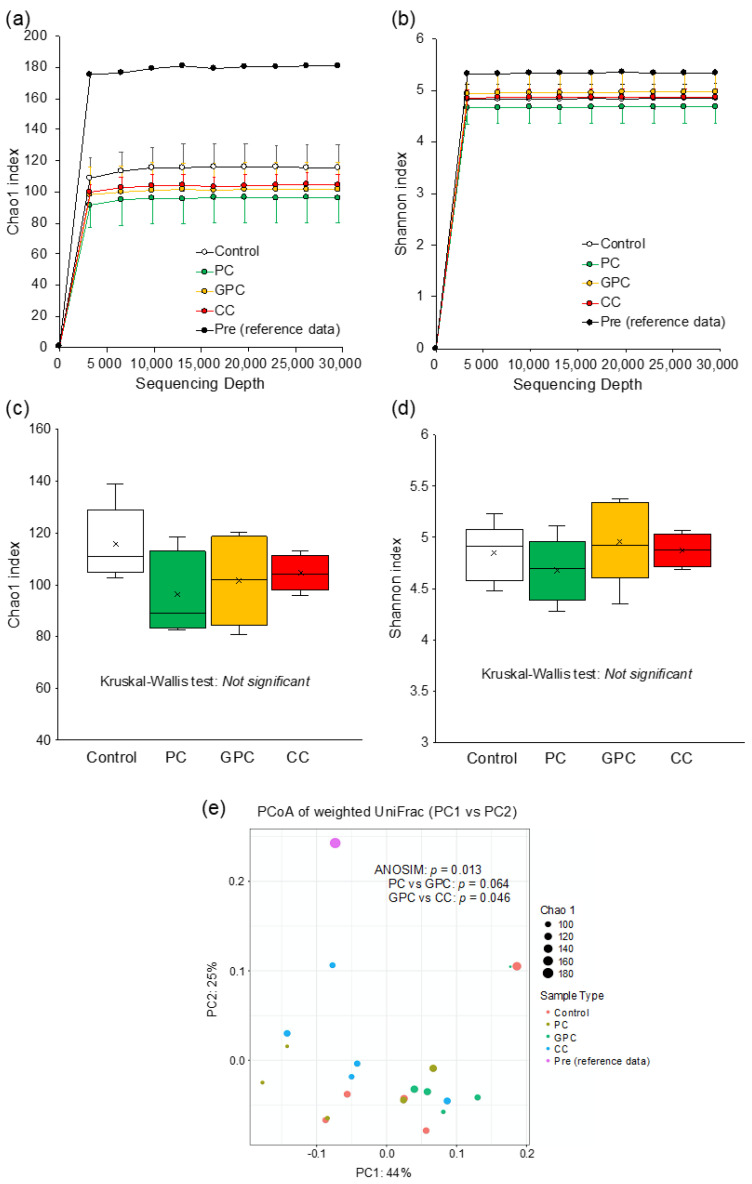
Effects of dietary choline compounds on diversity of fecal microbiota. (**a**,**b**) Bacterial rarefaction curves based on the α Chao 1 and Shannon diversity indices at the genus level, respectively. (**c**,**d**) Boxplots based on the α Chao 1 and Shannon diversity indices at the genus level, respectively. (**e**) Principal coordinate analysis (PCoA) based weighted UniFrac distance in β diversity at the genus level.

**Figure 4:**
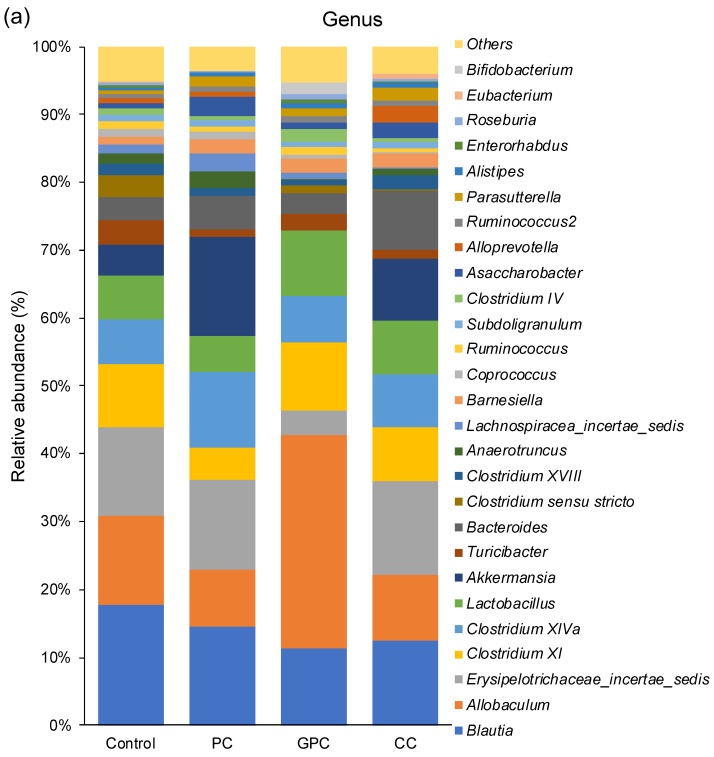

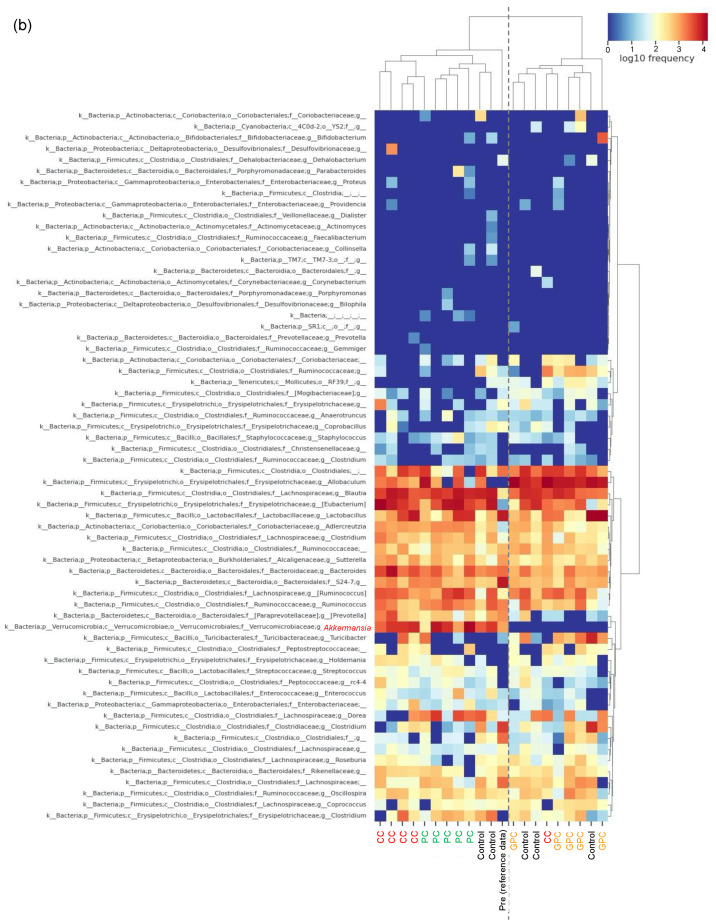
(**a**) Effects of dietary choline compounds on relative abundance of fecal microbiota at the genus level. The 27 dominant microbes with relative mean abundances of >0.5% are shown. Microbes with relative mean abundances of <0.5% are summed into “others.” (**b**) Heatmap and hierarchical clustering dendrogram of gut microbiota composition at the genus levels.

**Figure 5:**
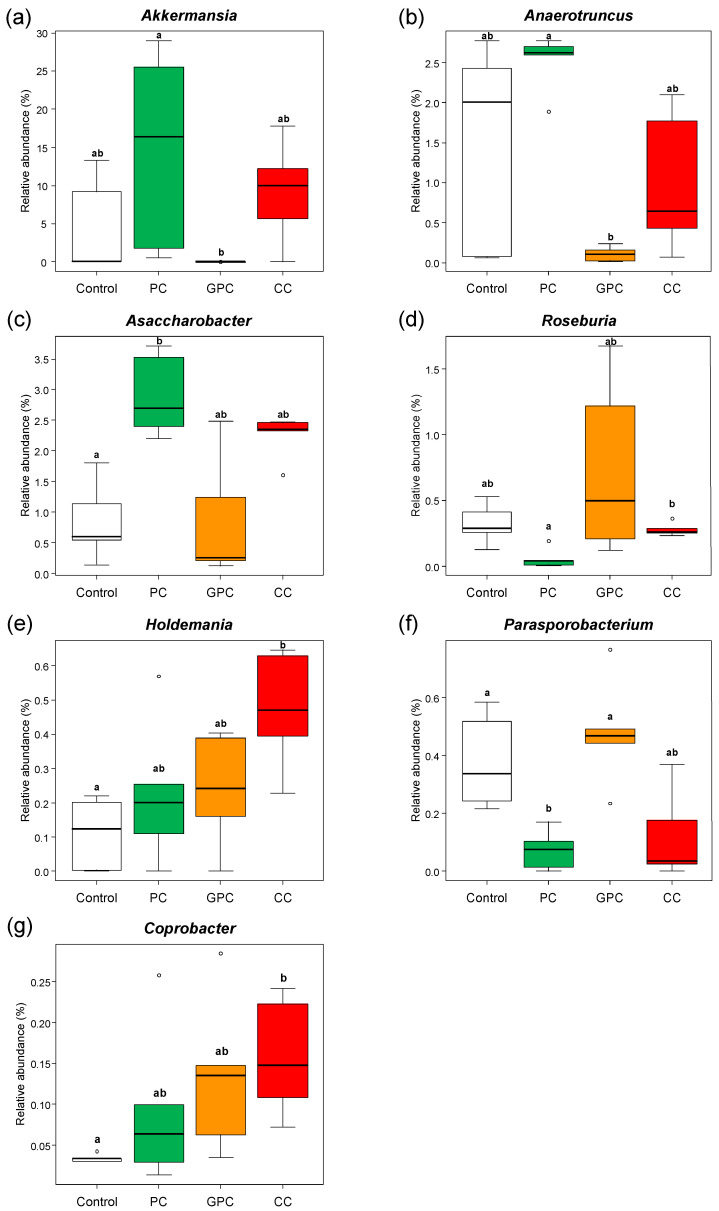
Effects of dietary choline compounds on relative abundances of fecal microbiota at the genus level. (**a**–**g**) Relative abundances of *Akkermansia*, *Asaccharobacter*, *Anaerotruncus*, *Roseburia*, *Holdemania*, *Parasporobacterium*, and *Coprobacter*, respectively. Data are represented as box plots with outliers (shown as white circles) (*n* = 5 per group). ^a,b^ Different letters show significant difference at *p* < 0.05 using the Kruskal–Wallis test, followed by Steel–Dwass multiple comparison post hoc tests.

To gain additional insight into the reason why dietary PC does not affect plasma TMAO levels, whereas dietary GPC and CC both increase it, here we evaluated fecal microbiota in rats using 16S rRNA amplicon sequencing. Figure 3 shows the α and β diversity results of the fecal microbiota. The rarefaction curves based on the α Chao 1 and Shannon diversity indices demonstrated good sequencing depth, as plateaus were reached in all samples after approximately 30,000 reads (Figure 3a,b). Box plots based on the α Chao 1 (Figure 3c) and Shannon (Figure 3d) diversity indices at the genus level showed that there were no significant differences among the four groups. Figure 3e shows the PCoA plot based on the weighted UniFrac distance at the genus level. The fecal microbiota structure of the PC group tended to differ (*p* = 0.064) from that of the GPC group. Moreover, the fecal microbiota structure of the GPC group differed from that of the CC group (*p* = 0.046). The taxonomic assignment at the genus level identified a total of 176 bacterial genera. Furthermore, the 27 dominant microbes (with relative mean abundances of >0.5%) accounted for ~95% of the total abundance (Figure 4a). The heatmap and the hierarchical clustering dendrogram of gut microbiota composition at the genus level both clearly show that the gut microbiota profiles of the PC and GPC groups differed; this difference was driven by the relative abundance of *Akkermansia* (Figure 4b). Regarding the relative abundances of each microbe at the genus level among the four groups, the PC group had significantly higher relative abundances of *Akkermansia* (Figure 5a) and *Anaerotruncus* (Figure 5b) than the GPC group. The PC group, meanwhile, had a significantly higher relative abundance of *Asaccharobacter* (Figure 5c) than the control group, and a lower relative abundance of *Parasporobacterium* (Figure 5f). Moreover, the PC group had a significantly lower relative abundance of *Roseburia* (Figure 5d) than the CC group. Furthermore, the CC group had significantly higher relative abundances of *Holdemania* (Figure 5e) and *Coprobacter* (Figure 5g) than the control group. A previous study into ApoE^−/−^ mice showed that the oral supplementation of *Akkermansia muciniphila* attenuated atherosclerotic lesions by ameliorating metabolic endotoxemia-induced inflammation by restoring the gut barrier function [21]. Furthermore, several studies conducted on human subjects have demonstrated the beneficial effects of *A. muciniphila* against obesity, type 2 diabetes, and hypertension [22,23,24,25]. Taken together, we suggest that the beneficial effects of dietary PC are partly attributable to the resulting increase in the relative abundance of *A. muciniphila* in the gut microbiota.

**Figure 6:**
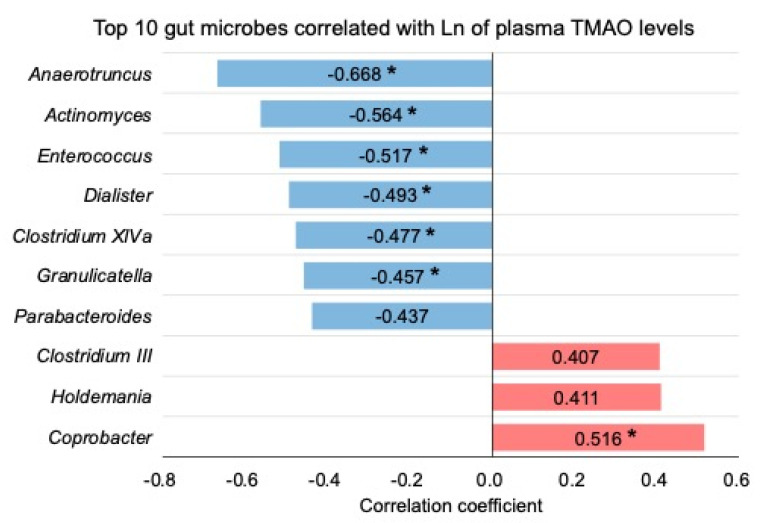
Top 10 gut microbes correlated with logarithmic-transformed TMAO plasma levels. Asterisk shows significant difference at *p* < 0.05. “LN” indicates a natural logarithm.

As shown in Figure 6, we found a positive association between plasma TMAO levels and the relative abundance of *Coprobacter*; we also discovered inverse associations between plasma TMAO levels and the relative abundances of *Anaerotruncus*, *Actinomyces*, *Enterococcus*, *Dialister*, *Clostridium* XIVa, and *Granulicatella*. *Anaerotruncus*, *Enterococcus*, *Dialister*, *Clostridium* XIVa, and *Granulicatella* belonging to the *Firmicutes* phylum, *Actinomyces* to the *Actinobacteria* phylum, and *Coprobacter* to the *Bacteroidetes* phylum, respectively. Although several species belonging to the *Firmicutes*, *Actinobacteria*, and *Proteobacteria* phyla can potentially produce TMA (TMA is the precursor of TMAO) [26], the taxonomic levels of family and genus in the species described above were different from those of the seven microbes found in this study. A significant negative correlation has been discovered between the relative abundances of *Anaerotruncus* (at the genus level) and *Enterococcus gallinarum* (at the species level) and fecal TMA levels [27]. This supports our findings, to some extent. During the conversion of choline to TMA by gut microbiota in the host, the key step in the microbial catabolism of choline is the cleavage of its C-N bond; this step is catalyzed by CutC and CutD [15,16]. Therefore, it is possible that *Anaerotruncus* and *Enterococcus* act on TMA-producing bacteria to indirectly suppress TMA production. Alternatively, they could produce active substances that suppress the activities of CutC and CutD, thereby directly suppressing TMA production in the gut. However, no previous studies have obtained findings that support our results for the five microbes that were found to correlate with plasma TMAO levels. Therefore, further studies are needed to elucidate the mechanism by which the observed changes in the relative abundances of these five microbes lead to changes in plasma TMAO levels. In addition to the changes in TMA production through the changes in the gut microbiota by dietary GPC and CC, it is desirable to examine whether they affect hepatic FMO1 and FMO3 protein levels.

**Figure 7:**
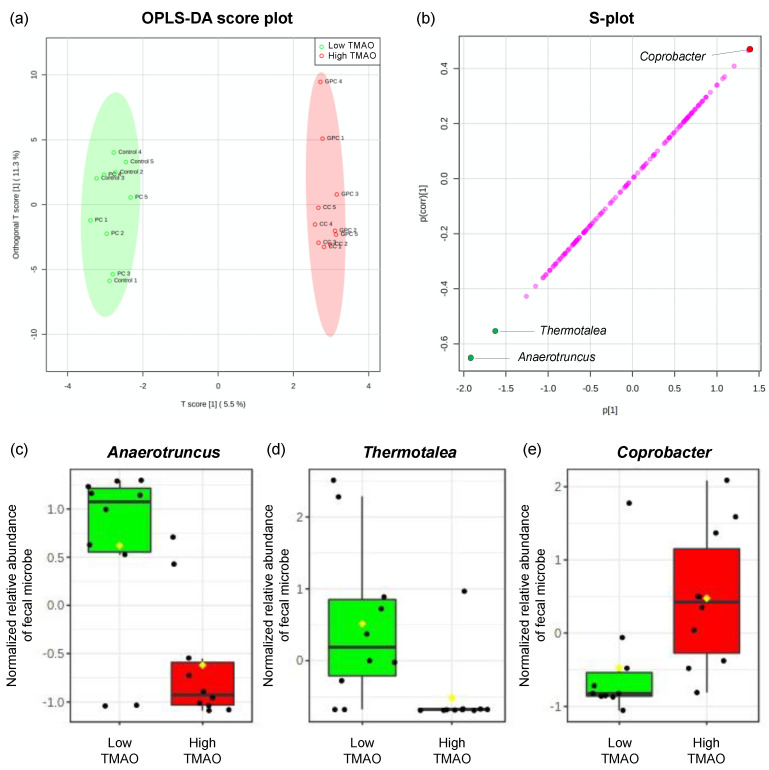
OPLS-SA of low and high TMAO groups, based on fecal microbiota at the genus level. (**a**) OPLS-DA score plot, (**b**) its corresponding S-plot, and (**c–e**) characteristic gut microbes that can discriminate between the low and high TMAO groups (normalized data are represented as box plots with each and mean values as shown black and yellow squares, respectively). Variables with a variable importance of projection (VIP) of >2.0 are highlighted with green or red filled circles.

Here, we tried to identify predictors of plasma TMAO levels using stepwise multiple regression analysis; we identified seven microbes that were correlated with plasma TMAO levels. As a result, we obtained a good multiple regression model for plasma TMAO levels, with adjusted R^2^ values of >0.5 (Table 2). This model showed VIF values of <5 and a Durbin–Watson ratio close to 2. The adjusted R^2^ value of this model was 0.656 (*p* < 0.001), and the relative abundances of *Anaerotruncus* and *Coprobacter* (in decreasing order of absolute value of β coefficient) both contributed to plasma TMAO levels (Table 2). Furthermore, using OPLS-DA, we attempted to search for gut microbes that could help to distinguish between the low (control and PC groups) and high (GPC and CC groups) TMAO groups. As shown in Figure 7a, the low and high TMAO groups were clearly divided into two groups, and the variables with VIP > 2.0 were (in decreasing order) *Anaerotruncus*, *Thermotalea*, and *Coprobacter* (Figure 7b,c). In particular, *Anaerotruncus* and *Coprobacter* were located at both ends of the OPLS-DA-corresponding S-plot, and so were considered to contribute strongly to discriminating between the two groups. This finding was consistent with the results of the multiple regression analysis. According to the previous report [26], however, TMA production potential appears to be absent in the microbes belonging to the *Bacteroidetes* phylum. Therefore, further studies are needed to evaluate whether *Coprobacter* belonging to the *Bacteroidetes* phylum can generate TMA from GPC and CC.

## 3. Materials and Methods

### 3.1. Animals and Diets

All animal experiments were conducted in accordance with the Guidelines for Animal Experiments of the University of Nagasaki, Siebold, and Law no. 105, and Notification no. 6 of the Government of Japan. The animal protocol in this study was approved by the Institutional Review Board of the University of Nagasaki, Siebold (authorization no. R02-10). Five-week-old male Sprague-Dawley (SD) rats (Jcl:SD) were purchased from CLEA Japan, Inc. (Osaka, Japan). The rats were housed individually in metal cages, in an air-conditioned room at 22 ± 1 °C with 55 ± 5% humidity, under a 12 h light-dark cycle. After an eight-day adaptation period on a powder chow diet (CE-2; CLEA Japan, Inc.), 20 rats were assigned to one of four groups: control, PC, GPC, and CC (*n* = 5 per group). Experimental diets were prepared according to the AIN-76 formula containing a high sucrose content [28], with several modifications. The details of the experimental diet composition are presented in Table 3. The rats were allowed free access to the diet and water for four weeks. Feces were collected for 48 h during the adaptation period, and at the end of the experiment. At the end of the four-week feeding period and after a 6 h starvation period, blood was collected from the abdominal vein of each rat using a syringe. The ethylenediaminetetraacetic acid (EDTA)-containing plasma was prepared by centrifugation at 1200 × g for 15 min at 4 °C. The liver, soleus muscle, and abdominal (epididymal, perirenal, and mesenteric) white adipose tissues (WATs) were immediately excised and weighed within 4 h. The collected samples were stored at −80 °C until further analysis.

### 3.2. Analysis of Plasma Biochemical Parameters

Plasma levels of TG, total cholesterol, PLs, non-esterified fatty acids (NEFA), and glucose were measured using commercial enzyme assay kits (TG E-test, Wako; Cholesterol E-test, Wako; Phospholipid C-test, Wako; NEFA C-test, Wako; Glucose CII-test, Wako; FUJIFILM Wako Pure Chemical Co., Osaka, Japan).

### 3.3. Analysis of Hepatic Phospholipids Contents

Total lipids from the liver were extracted using the Bligh and Dyer method [29], with slight modifications. Briefly, frozen liver tissue was homogenized with 0.1 M KCl solution and mixed with methanol (containing 0.01% butylated hydroxytoluene) and chloroform at a ratio of 1:1:1. The homogenate was centrifuged at 880× *g* for 15 min at room temperature. The lower organic phase containing the extracted lipids was collected three times and then dried under nitrogen gas. The extracted lipids were dissolved in 2-propanol. Hepatic PL content was then measured according to the method of Rouser et al. [30].

### 3.4. Analysis of Plasma TMAO Levels

Sample preparation for the quantitative analysis of TMAO in plasma was performed according to the method described by Ufnal et al. [31]. Plasma TMAO levels were measured using liquid chromatography (LC) coupled with triple-quadrupole mass spectrometry (MS; LCMS-8050; Shimadzu, Kyoto, Japan). The LC-MS conditions were as follows: solvent A, 0.2% aqueous formic acid; solvent B, methanol with 0.2% formic acid; column, Discovery HS F5-3 (Supelco Analytical, Bellefonte, PA, USA); column temperature, 40 °C; flow rate, 0.3 mL·min^−1^; gradient program, 0–2 min: %B = 0, 2–6 min: %B = 0–15 gradient, 6–6.5 min: %B = 15–100 gradient, 6.5–9.5 min: %B = 100, and 9.5–10 min: %B = 100–0 gradient. MS spectra were obtained in the electrospray ionization (ESI) positive ion mode. The temperatures of the ESI interface, desolvation line, and heat block were 300, 250, and 400 °C, respectively. Nebulizer, heating, and drying nitrogen gas flows were set to 3.0, 10, and 10 L·min^−1^, respectively. A multiple reaction monitoring (MRM) transition of *m/z* 76.08 > 58.1 was used. The dwell time was set at 10 ms for the transition.

### 3.5. Analysis of Hepatic Messenger Ribonucleic Acid (mRNA) Levels

Total RNA was extracted from frozen liver tissue using RNAzol^®^ RT reagent (Molecular Research Center, Inc., Cincinnati, OH, USA); it was then converted into complementary deoxyribonucleic acid (cDNA) using PrimeScript^™^ RT Master Mix (Perfect Real Time) (Takara Bio Inc., Shiga, Japan), according to the manufacturer’s instructions. Polymerase chain reaction (PCR) amplification was performed in a final volume of 20 µL, which contained SYBR Green (THUNDERBIRD^®^ SYBR^®^ qPCR Mix; Toyobo Co., Ltd., Osaka, Japan), 0.3 µM of each primer (Fasmac Co., Ltd., Kanagawa, Japan), and 20 ng of cDNA; a real-time PCR system was used (LightCycler^®^96; Nippon Genetics Co., Ltd., Tokyo, Japan). The reaction conditions were as follows: hot start at 95 °C for 60 s, followed by 45 cycles of denaturation at 95 °C for 15 s and annealing/extension at 60 °C for 45 s. Relative mRNA levels were determined using the ΔΔCT method [32], using β-Actin (*Actb*) as a normalization control. The primers used were as follows: flavin-containing dimethylaniline monooxygenase 1 (*Fmo1*, forward: 5′-AGAGACACAAGCTCGATGGG-3′, reverse: 5′-GCACAAGCCAAATCCGCTAT-3′), *Fmo3* (forward: 5′-AGGAGCCAGGAACATGGAAAG-3′, reverse: 5′-GGAGCTTATGATGACCTGCTGA-3′), and *Actb* (forward: 5′-TCAGGTCATCACTATCGGCA-3′, reverse: 5′-TCATGGATGCCACAGGATTC-3′).

### 3.6. Analysis of Gut Microbiota in Rats Fed Each Choline Compound

DNA extraction was conducted according to a previously described method [33]. DNA was extracted using an automated DNA isolation system (GENE PREP STAR PI-480 KURABO, Japan). The V3-V4 regions of bacterial and archaeal 16S ribosomal RNA (rRNA) were amplified using the Pro341F/Pro805R primers and dual-index method [33,34]. Barcoded amplicons were paired-end sequenced on a 2×284-bp cycle using the MiSeq system with MiSeq Reagent Kit version 3 (600 cycle) chemistry. Paired-end sequencing reads were merged using the fastq-join program (Expression Analysis, a Quintiles Company, Durham, NC, USA), with default settings [35]. Only joined-reads that had a quality value score of ≥20 for more than 99% of the sequence were extracted; this was performed using the FASTX-Toolkit [36]. The chimeric sequences were deleted using usearch6.1 (https://drive5.com/usearch/, accessed on 18 March 2021) [37,38]. Sequence analysis of the sequence reads was performed manually using the Ribosomal Database Project (RDP) Multiclassifier tool v. 2.11, which is available from the RDP website (http://rdp.cme.msu.edu/classifier/, accessed on 18 March 2021) [39]. Bacterial and Archaeal species identification from sequences was performed using the Metagenome@KIN v. 2.2.1 analysis software (World Fusion, Tokyo, Japan) and the TechnoSuruga Lab Microbial Identification database DB-BA v. 13.0 (TechnoSuruga Laboratory, Shizuoka, Japan), with a homology of ≥97% [40]. The joined amplicon sequence reads were processed using Quantitative Insights Into Microbial Ecology version 2 (QIIME2) v. 2020.6 (https://qiime2.org/, accessed on 18 March 2021) [41]. Sequences with quality value scores of <33 and chimeric sequences were filtered using Divisive Amplicon Denoising Algorithm 2 (DADA2) denoise-single plugin v. 2017.6.0 (https://benjjneb.github.io/dada2/index.html, accessed on 18 March 2021) [42]. Taxonomy was assigned using the Greengenes database v. 13.8 (http://greengenes.lbl.gov, accessed on 18 March 2021), based on an average percent identity of 99% [43], by training a Naive Bayes classifier using the q2-feature-classifier plugin (https://github.com/qiime2/q2-feature-classifier, accessed on 18 March 2021). Alpha diversity indices (Chao1 and Shannon indices) at the genus level were calculated using the alpha-rarefaction plugin. Beta diversity at the genus level was visualized using principal coordinate analysis (PCoA); it was analyzed using weighted UniFrac distance using the core-metrics-phylogenetic plugin. Heatmap and hierarchical clustering at the genus level were shown using the feature-table heatmap plugin.

### 3.7. Statistical Analysis

All values except for fecal microbiota data are expressed herein as means ± standard error of the mean (SEM). All data were analyzed using the Shapiro–Wilk test as a normality test. When the data were recognized as having a non-normal distribution, they were normalized by logarithmic transformation and then re-analyzed using the Shapiro–Wilk test. Parametric tests were used for further statistical analysis, because the normality of all data was confirmed. Comparisons among the four groups were performed using one-way analysis of variance (ANOVA), followed by the Tukey–Kramer multiple comparison post hoc test. Data of fecal microbiota at the genus level are expressed herein using box and whisker plots. The statistical significances of the Chao1 and Shannon indices, based on the relative abundances of fecal microbiota at the genus level among the four groups, were assessed using the Kruskal–Wallis test. The statistical significance of the weighted UniFrac distance, based on the relative abundances of fecal microbiota at the genus level among the four groups, was assessed by the analysis of similarities (ANOSIM) test. The relative abundances of fecal microbiota at the genus level were assessed using the Kruskal–Wallis test, followed by the Steel–Dwass multiple comparison post hoc test. Spearman correlation coefficients were used to determine the significances of the associations between plasma TMAO levels and the relative abundances of fecal microbiota at the genus level. Stepwise multiple regression analysis was performed to explore the determinants (relative abundances of fecal microbiota at the genus level) of plasma TMAO levels. Fecal microbiota that were found to be significantly correlated with plasma TMAO levels were included as predictors in the original model. A *p* value of <0.01 and an adjusted R^2^ value of >0.5 were considered to imply significance for the purpose of regression analysis. The variance inflation factor (VIF) was used to detect multicollinearity between the independent variables of the model [44]. A VIF of <5 was considered acceptable. The Durbin–Watson ratio was used to detect the presence of autocorrelation in the model. A Durbin–Watson ratio close to 2 was considered acceptable. The normality of the residuals in the multiple regression model was assessed using the Shapiro–Wilk test. Blood TMAO levels have also been used as biomarkers for coronary artery disease; they are considered to be low risk at concentrations of <6.2 µM [45]. Therefore, we divided the rats into two groups (low and high TMAO groups), with 6.2 µM as the cut-off value; we then performed orthogonal partial least squares discriminant analysis (OPLS-DA) based on the relative abundances of fecal microbiota at the genus level. Differences were considered to be significant at *p* < 0.05. Statistical analysis was performed using EZR, which is a graphical user interface for R (The R Foundation for Statistical Computing, Vienna, Austria, version 4.0.4) [46], and SPSS v. 28 software for Windows (IBM Japan, Ltd., Tokyo, Japan). OPLS-DA was performed using the free web-based software MetaboAnalyst 5.0 (accessed on 13 December 2021) [47].

## 4. Conclusions

In summary, our results clearly indicate that dietary PC, unlike GPC and CC, does not increase plasma TMAO levels in rats. We identified several gut microbes that were associated with changes in plasma TMAO levels in rats that were fed choline compounds. Among these, the relative abundances of *Anaerotruncus* and *Coprobacter* may have large effects on changes in plasma TMAO levels. Our data will be valuable as additional baseline data, and for the quantitative comparison of dietary choline compounds regarding in vivo TMAO production.

## Figures and Tables

**Table 1:** Effects of diets containing choline compounds on morphometric and metabolic parameters in rats.

	Control	PC	GPC	CC
Initial body weight (g)	203 ± 5	206 ± 2	206 ± 2	205 ± 4
Final body weight (g)	420 ± 10	432 ± 4	422 ± 10	428 ± 7
Food intake (g·d^−1^)	24.4 ± 0.6	24.2 ± 0.4	24.5 ± 0.1	24.2 ± 0.5
Organ weights (g per 100 g of body weight)				
Liver	4.15 ± 0.17	4.41 ± 0.1	4.15 ± 0.27	4.23 ± 0.12
Soleus muscle	1.04 ± 0.07	1.08 ± 0.06	1.07 ± 0.03	1.01 ± 0.03
White adipose tissue (WAT) weights (g per 100 g of body weight)				
Epididymal	1.75 ± 0.10 ^a^	1.56 ± 0.05 ^ab^	1.45 ± 0.09 ^ab^	1.4 ± 0.07 ^b^
Perirenal	2.26 ± 0.17 ^a^	1.69 ± 0.15 ^ab^	1.52 ± 0.21 ^b^	1.77 ± 0.19 ^ab^
Mesenteric	1.32 ± 0.12	1.19 ± 0.06	1.09 ± 0.08	1.05 ± 0.1
Abdominal *	5.33 ± 0.36	4.44 ± 0.19	4.07 ± 0.35	4.21 ± 0.34
Plasma levels				
TG (mg·dL^−1^)	189 ± 36	227 ± 27	164 ± 44	211 ± 50
NEFA (mEq·dL^−1^)	0.404 ± 0.032	0.384 ± 0.019	0.363 ± 0.053	0.388 ± 0.02
Total cholesterol (mg·dL^−1^)	77.3 ± 7.9	76.3 ± 7	93.5 ± 10	76.9 ± 3.4
PL (mg·dL^−1^)	138 ± 30	104 ± 11	149 ± 24	117 ± 18
Glucose (mg·dL^−1^)	259 ± 32	232 ± 9	237 ± 15	241 ± 13
Liver PL contents (mg per whole liver)	476 ± 33 ^a^	409 ± 20 ^ab^	358 ± 18 ^b^	378 ± 13 ^b^
mRNA levels of enzymes related to TMA metabolism in the liver				
*Fmo1* (arbitrary unit)	1 ± 0.21	1.38 ± 0.2	1.04 ± 0.19	1.03 ± 0.26
*Fmo3* (arbitrary unit)	1 ± 0.16	0.894 ± 0.159	0.798 ± 0.158	0.737 ± 0.107
Feces				
Weight (wet g·d^−1^)	2.32 ± 0.11	2.2 ± 0.12	2.3 ± 0.16	2.26 ± 0.14

* Abdominal WAT weights were calculated by adding the weights of epididymal, perirenal, and mesenteric WAT. Values are expressed as means ± standard error of the mean (SEM; *n* = 5 per group). ^a,b^ Different superscript letters show significant differences at *p* < 0.05, as determined using one-way analysis of variance (ANOVA) followed by Tukey–Kramer multiple comparison post hoc tests.

**Table 2:** Best predictors of variations in plasma TMAO levels from stepwise forward regression analysis.

Response Variable	Predictor Variable (Fecal Microbe)	β Coefficient	*p* Value	VIF	Durbin-Watson Ratio	Normality of Unstandardized Residual	Adjusted R^2^
LN plasma TMAO	*Anaerotruncus*	−0.725	*p* < 0.001	1.000	1.393	*Yes*	0.656 (*p* < 0.001)
	*Coprobacter*	0.401	*p* < 0.01	1.000			

“*Yes*” indicates normal distribution of residual. “LN” indicates a natural logarithm. VIF, variance inflation factor.

**Table 3:** Composition of experimental diets.

	Control	PC	GPC	CC
Ingredients	(g·kg^−1^ diet)			
Sucrose	479	474	472.364	475.398
Casein	200	200	200	200
β-Cornstarch	150	150	150	150
Cellulose	50	50	50	50
Soybean oil ^a^	70	55	70	70
Soybean PC ^b^	---	20	---	---
GPC ^c^	---	---	6.636	---
Choline chloride (CC) ^d^	---	---	---	3.602
Mineral mixture (AIN-76)	35	35	35	35
Vitamin mixture (AIN-76)	10	10	10	10
DL-Methionine	3	3	3	3
Choline bitartrate	2	2	2	2
Cholesterol	1	1	1	1
	(mmol·kg^−1^ diet)			
Total choline	7.9	33.7	33.7	33.7
Total fatty acids	237.6	238.3	237.6	237.6

^a^ Average M.W. = 884; Nacalai Tesque, Inc., Kyoto, Japan. ^b^ The purity of PC was ≥94% (average M.W. = 775.04; EMD Millipore Corp., Darmstadt, Germany). ^c^ The purity of GPC was ≥98% (M.W. = 257.22; Tokyo Chemical Industry Co., Ltd., Tokyo, Japan). ^d^ The purity of CC was ≥98% (M.W. = 139.62; Tokyo Chemical Industry Co., Ltd., Tokyo, Japan).

## Data Availability

The data presented in this study are available on request from the corresponding author. The data are not publicly available due to have not set up a public archive platform for data sharing.
